# MiR-204 regulates cardiomyocyte autophagy induced by ischemia-reperfusion through LC3-II

**DOI:** 10.1186/1423-0127-18-35

**Published:** 2011-06-01

**Authors:** Jian Xiao, Xiaoyan Zhu, Bin He, Yufeng Zhang, Bo Kang, Zhinong Wang, Xin Ni

**Affiliations:** 1 Department of Cardiothoracic Surgery, Changzheng Hospital, the second military medical university, Fengyang road 415#, shanghai, China, 200003; 2 Department of Physiology, the second military medical university, Xiangyin road 800#, Shanghai, China, 200433; 3 Department of Anesthesiology, Xinhua Hospital, Shanghai Jiaotong University School of Medicine, Kongjiang road 1665#, Shanghai, China, 200092

## Abstract

**Background:**

Autophagy plays a significant role in myocardial ischemia-reperfusion (IR) injury. So it is important to inhibit autophagy to protect cardiomyocytes besides anti-apoptosis. MiRNA has been demonstrated to protect cardiomyocytes against apoptosis during IR, while whether it has anti-autophagy effect has not been known. The aim of this study was to investigate whether miR-204 regulated autophagy by regulating LC3-II protein, which is the marker of autophagosome during myocardial IR injury.

**Methods:**

Adult SD rats were randomized to Control and IR groups. IR group was treated with 30 min ischemia by ligating the left anterior descending coronary artery, followed by 2 h reperfusion by loosing the ligation. The expression of miR-204 was measured by RT-PCR, and LC3 protein was measured by western-blot.

**Results:**

We found that IR induced cardiomyocytes autophagy, together with down-regulation of miR-204 and up-regulation of LC3-II protein. And, we have found that LC3-II protein was regulated by miR-204, using the method of transferring miR-204 mimic or AMO-204 into the cardiomyocytes, before.

**Conclusions:**

These studies provided evidence that miR-204 played an important role in regulating autophagy through LC3-IIprotein during IR.

## Background

Autophagy is a type of programmed cell death. It has been suggested to be essential for cell homeostasis [1-3]. It can determine the cell survival together with apoptosis and necrosis [4,5]. Autophagy level is very low in physiological conditions, and is upregulated in many pathophysiological processes [6,7]. Because cardiomyocytes are terminally differentiated cells which can not divide again, suitable autophagy is essential for the maintenance of cardiomyocytes homeostasis. So, autophagocytic deficiencies or excess is associated with many cardiac pathologies, such as ischemia, IR, and heart failure [8,9]. It has been found that autophagy increased after IR [10], but it is still unclear whether autophagy protects the heart against IR injury or contributes to cell death. It seems that modest levels of autophagy appear to be protective. While high levels of autophagy may cause self-digestion and promote cell death [11].

Autophagy is regulated by many autophagy related genes (Atgs) which are involved in autophagosome formation [12]. Among these Atgs, LC3 (microtubule-associated protein 1 light chain 3, Atg8) is localized on the autophagosome membrane. So LC3 is essential for the formation of autophagosome [13]. During the formation of autophagosome, the soluble form of LC3 (LC3-I) is convered to the autophagic vesicle-associated form (LC3-II), which is an important marker of autophagy [14]. So it is possible to control the process of autophagy by up-regulating or down-regulating LC3, and the molecular mechanism for this effect has yet to be elucidated.

As we know, microRNAs (miRNAs or miRs), which negatively regulate protein expression in diverse biological and pathological processes, have been demonstrated to play an important role in myocardial injury [15-17]. It has been observed that many miRNAs regulate cell apoptosis, such as miR-1, miR-133, miR-199, miR-208, miR-320, miR-21, and miR-204, etc [18-23]. However, it is well known that when apoptosis is blocked, the cells, which preferentially die by apoptosis, may die by autophagy [24]. So it will be beneficial for cell survival if autophagy is inhibited together with apoptosis. We found that miR-204, which has anti-apoptosis effect, may also regulate LC3 expresion through the 9 complementary bases, according the bioinformatics of Targetscan.

So the present study was undertaken to see whether miR-204 was dysregulated by ischemia-reperfusion (IR), and if it may inhibit autophagy during hypoxia-reoxygenation by regulating LC3.

## Material and methods

### Animal care

All animal experiments were approved by the Animal Research Ethics Committee of the Second Military Medical University, Shanghai, China. The investigation conformed with the guide for the care and use of laboratory animals published by the US National Institutes of Health.

### IR model and experimental protocols

SD rats (250-300 g) were anesthetized with 10% chloral hydrate (300 mg/kg, i.p.) before endotracheal intubation. IR was induced by ligating the left anterior descending artery (LAD) for 30 min, followed by loosening the ligature for 120 min. Successful ligation of LAD was evidenced by immediate regional cyanosis in the anterior ventricular wall and the apex of the heart with color change greater than 40% of the left ventricle (LV) and confirmed by electrocardiography(ECG).

### Experimental protocols

Twenty rats were equally randomly assigned into two groups: Control group (Con group, n = 10), where the rats underwent thoracotomy without ligation; IR group (n = 10), where the rats were treated with ischemia for 30 min and reperfusion for 120 min.

### Infarct size measurement

Infarct size of the myocardium was measured as previously described. Infarct area (INF) and area at risk (AAR) were determined by computerized planimetry. The percentage of INF/AAR was calculated.

### LDH assay

Blood serum was collected after 180 min reperfusion for determination of lactate dehydrogenase (LDH).

### Quantitative real-time RT-PCR of miR-204

Total RNA of cells was isolated by using TRIzol reagent, and reverse transcribed according to the manufacturer's instructions (Fermentas, in CA). The annealing temperature of miRNA-204 was set at 60°C. The comparative Ct (threshold cycle) method with arithmetic formulae (2^-ΔΔCt^) was used to determine relative quantitation of gene expression of both target and housekeeping genes (β-actin). The primers of miR-204 used in the study are shown in Table 1.

**Table 1:** Primers used for quantitative real-time RT-PCR

Type	Name	Sequence
RT-primer	miR-204	5'- GTCGTATCCAGTGCAGGGTCCGAGGTA TTCGCACTGGATACGACCCTTCTAGGCAT-3'
PCR-primer	miR204-F	5'-CTGTCACTCGAGCTGCTGGAATG-3'
	miR204-R	5'-ACCGTGTCGTGGAGTCGGCAATT-3'
	β actin-R	5'-ATGGTGGGTATGGGTCAGAAGG-3'
	β actin-F	5'-TGGCTGGGGTGTTGAAGGTC-3'

### Western-blotting

Protein concentration was determined with BCA protein assay kit according the manufacturer's protocol. Equal amounts of protein (60 ug) from the cardiomyocytes were subjected to Western-blotting analysis to evaluate LC3 expression with ECL detection kit (Amersham Biosciences, Piscataway, NJ). LC3 immunoreactivity was detected using a rabbit antiserum specific for rat Beclin1 protein (Sigma, USA) as primary antibodies. Detection of antigen-antibody complex formation was performed with horseradish peroxidase (HRP)-conjugated goat anti-rabbit secondary antibody. The LC3 concentrations were normalized with β-actin.

### Statistical analysis

Quantitative data are presented as mean ± standard error. Statistical significance was determined using T test or one-way ANOVA. P < 0.05 was considered statistically significant.

## Results

### Myocardium injury was induced by IR

**Figure 1:**
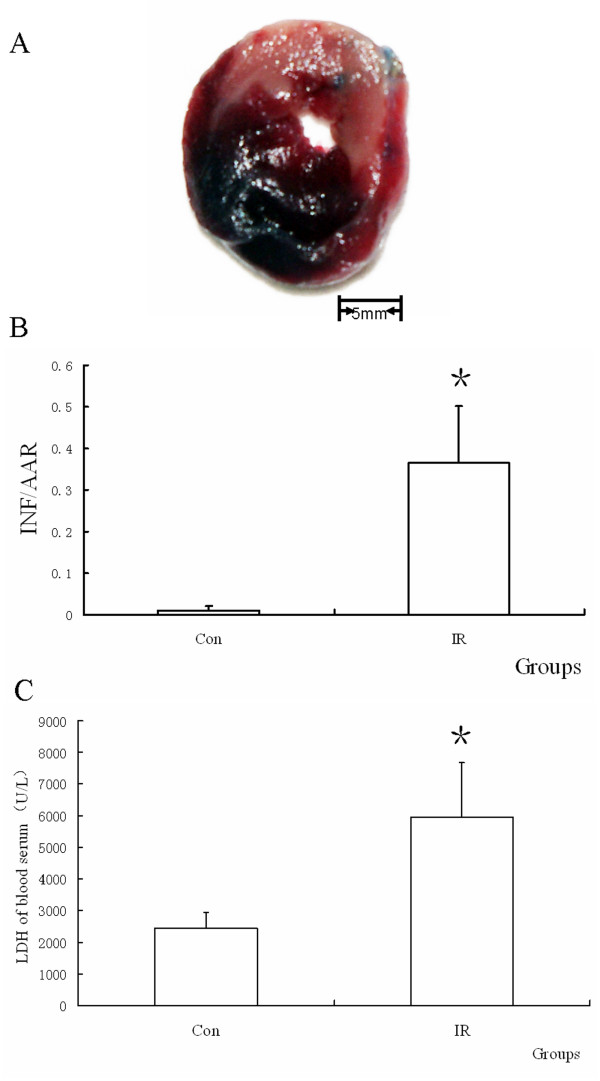
**The heart injury induced by IR**. (A) Representative mid-myocardial crosssections of TTC-stained hearts for IR. Dark blue area, nonischemic zone; red area, area at risk, AAR; white area, infracted tissue. (B) The ratio of INF/AAR. It was found that IR increased the relative INF size compared with Con group. (n = 10, *P = 0.000 < 0.05, compared with Con group). (C) LDH assay of blood serum. The activitie of LDH was increased by IR compared with Con group. (n = 10, *P = 0.000 < 0.05, compared with Con group).

The extent of myocardial infarction was evaluated after reperfusion. Representative photographs of midventricular cross sections of evans blue and TTC-stained hearts were taken from Control and IR groups. INF/AAR and LDH were shown in Figure 1.

### IR decreased the expression of miR-204

**Figure 2:**
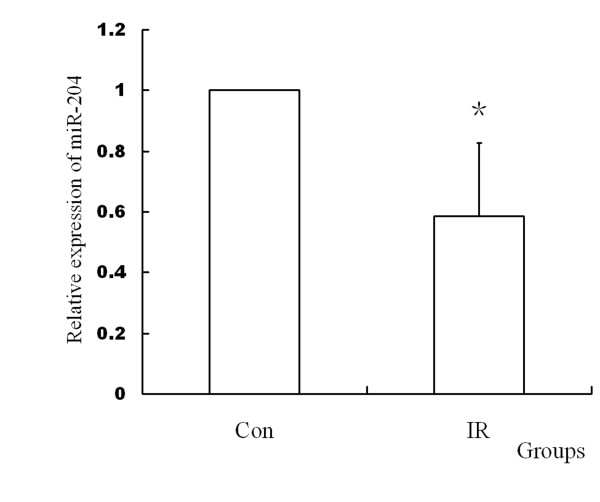
**Results of miR-204 expression with RT-PCR after IR injury**. It was found that miR-204 was down-regulated by IR. (n = 10, P = 0.026 < 0.05)

To demonstrate the effect of IR on miR-204, we compared the miR-204 between the control group and IR group (n = 10). It was found that IR significantly decreased miR-204 with the method of Real-time PCR. (Figure 2)

### IR up-regulated the protein level of LC3-II

**Figure 3:**
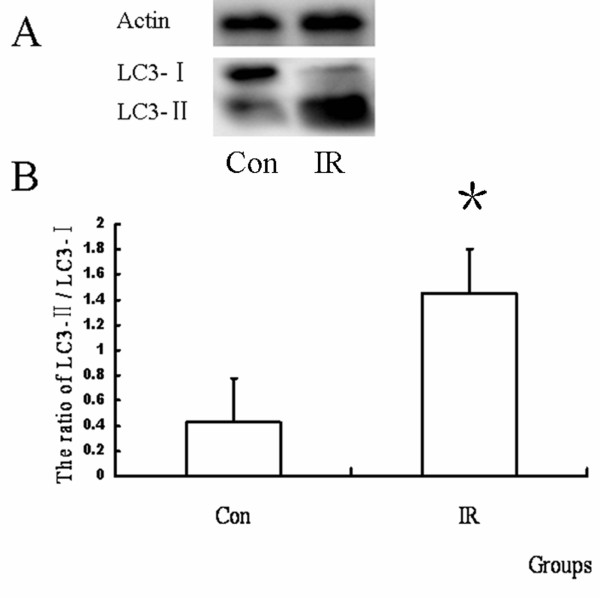
**Results of LC3 protein expression with western blot after IR injury**. (A) Representative western blot of LC3 from different groups. (B) The ratio of LC3-II/LC3-I in different groups. It was found that LC3-II was up-regulated by IR. (n = 10, *P = 0.000 < 0.05)

As a marker of autophagosome, the protein level of LC3-II represents the amount of autophagosome. So we compared the ratio of LC3-II/LC3-I between the control group and IR group (n = 10), and found that it was enhanced by IR. (Figure 3)

## Discussion

In recent years, autophagy has attracted great interest because it was involved in many physiological processes. If autophagy destroys the cytosol and organelles beyond a certain threshold, autophagic cell death will occur [25]. Autophagy was detrimental during reperfusion although it protected the cardiomyocytes during ischemia, which has been demonstrated by Matsui and his collaborators [26]. It has been reported that autophagy contributed to cell death when apoptosis is inhibited, and sometimes the early stages of autophagy were required for apoptosis [24]. LC3-II was the marker of autophagosome, instead of LC3-I. So the ratio of LC3-II/LC3-I could stand for the level of autophagy. In our study, we found that IR up-regulated the protein expression of LC3-II together with increasing the ratio of autophagy cell. So it would be beneficial for the revascularized hearts, to find a method of regulating LC3-II expression.

MiRNA is a group of small, non-coding RNAs which regulates gene expression in a sequence-dependent manner. They are endogenous regulators of gene expression, and have been demonstrated to be involved in cardiac IR injury. According to the bioinformatics of Targetscan, miR-204, which has anti-apoptosis effect [23], may regulate the expression of LC3-II.

In our study, it was found that IR could down-regulate miR-204 together with up-regulated LC3-II protein. When miR-204 mimic was transferred into cardiomyocytes, LC3-II protein was attenuated, and LC3-II protein was up-regulated by AMO-204 which was concentration- dependented as other miRNAs [27]. But LC3-I was not regulated by miR-204, as the previous study [28]. These results demonstrated that miR-204 may regulate cardiomyocytes autophagy through LC3-II during IR injury.

## Conclusions

Our results demonstrated that miR-204 played an important role by regulating LC3-II protein during IR. So it became possible to control the autophagy under a beneficial threshold by regulating miR-204 expression, for protecting the cardiomyocyte against IR injury. But it is still unknown how did IR regulated the expression of miR-204, and this will be our next work.

## Competing interests

The authors declare that they have no competing interests.

## Authors' contributions

Jian Xiao carried out the IR model, RT-PCR and Western-blot studies, and drafted the manuscript. Xiaoyan Zhu carried out the miR-204 transferring into cardiomyocytes, and participated in the sequence alignment of miR-204 mimic and AMO-204. Bin He carried out the primers synthesis of miR-204 and participated in drafting the manuscript. Bo Kang carried out the cardiomyocytes culture. Yufeng Zhang carried out the LDH assay. Zhinong Wang and Xin Ni conceived of the study, and participated in its design and coordination. All authors read and approved the final manuscript.
